# Neutralizing Antibody Responses to SARS-CoV-2 in Recovered COVID-19 Patients Are Variable and Correlate With Disease Severity and Receptor-Binding Domain Recognition

**DOI:** 10.3389/fimmu.2022.830710

**Published:** 2022-01-31

**Authors:** Agnieszka Katarzyna Maciola, Massimo La Raja, Monia Pacenti, Cristiano Salata, Giustina De Silvestro, Antonio Rosato, Giulia Pasqual

**Affiliations:** ^1^ Laboratory of Synthetic Immunology, Oncology and Immunology Section, Department of Surgery Oncology and Gastroenterology, University of Padua, Padua, Italy; ^2^ Department of Transfusion Medicine, Padua University Hospital, Padua, Italy; ^3^ Institute of Microbiology and Virology, Padua University Hospital, Padua, Italy; ^4^ Department of Molecular Medicine, University of Padua, Padua, Italy; ^5^ Oncology and Immunology Section, Department of Surgery Oncology and Gastroenterology, University of Padua, Padua, Italy; ^6^ Veneto Institute of Oncology IOV-IRCCS, Padua, Italy

**Keywords:** COVID-19, SARS-CoV-2, humoral response, neutralization, antibodies

## Abstract

Severe acute respiratory syndrome coronavirus type 2 (SARS-CoV-2) caused outbreaks of the pandemic starting from the end of 2019 and, despite ongoing vaccination campaigns, still influences health services and economic factors globally. Understanding immune protection elicited by natural infection is of critical importance for public health policy. This knowledge is instrumental to set scientific parameters for the release of “immunity pass” adopted with different criteria across Europe and other countries and to provide guidelines for the vaccination of COVID-19 recovered patients. Here, we characterized the humoral response triggered by SARS-CoV-2 natural infection by analyzing serum samples from 94 COVID-19 convalescent patients with three serological platforms, including live virus neutralization, pseudovirus neutralization, and ELISA. We found that neutralization potency varies greatly across individuals, is significantly higher in severe patients compared with mild ones, and correlates with both Spike and receptor-binding domain (RBD) recognition. We also show that RBD-targeting antibodies consistently represent only a modest proportion of Spike-specific IgG, suggesting broad specificity of the humoral response in naturally infected individuals. Collectively, this study contributes to the characterization of the humoral immune response in the context of natural SARS-CoV-2 infection, highlighting its variability in terms of neutralization activity, with implications for immune protection in COVID-19 recovered patients.

## Introduction

The severe acute respiratory syndrome coronavirus type 2 (SARS-CoV-2) is a viral pathogen first reported in China in December 2019 (1), which at the moment of writing has infected more than 260 million individuals worldwide, leading to more than 5 million deaths (2).

SARS-CoV-2 infection process starts with virus binding to angiotensin-converting enzyme 2 (ACE2) receptor on the host cells, with the Spike glycoprotein being the main factor mediating this mechanism. The protein gains fusion activity after proteolytic cleavage between two regions: S1 and S2. S1 contains the receptor-binding domain (RBD), whereas S2 contains the fusion peptide and the transmembrane domain anchoring the glycoprotein on the viral envelope (3).

In the context of COVID-19 infection, neutralizing antibodies targeting SARS-CoV-2 Spike are critical for several aspects. First, they can confer protection toward reinfection (4). Second, it has been shown that neutralizing responses in severe patients are associated with survival, highlighting the protective role of humoral response in disease resolution (5). Third, therapy based on the administration of monoclonal neutralizing antibodies decreases the risk of hospitalization and death in patients with mild-to-moderate symptoms, proving the beneficial effect of antibodies in preventing COVID-19 disease progression (6).

Analysis of the humoral response across multiple cohorts of COVID-19 recovered patients showed that SARS-CoV-2 natural infection can elicit neutralizing antibodies in the majority of cases, but accumulating evidence indicates that the magnitude of the response varies greatly across individuals (7, 8). This heterogeneity has been interpreted differently by public health policy across countries, resulting in different guidelines for the vaccination of COVID-19 recovered patients. To date, for instance in the United States, recovered patients are considered identical to naïve individuals for vaccination purposes, while in other European countries such as France and Italy, recovered patients are considered fully vaccinated with a single immunization. Similarly, the criteria that apply to COVID-19 recovered patients for the release of the “immunity pass” differ across European countries that adopted this type of certificate. Further, characterizing the variation of the neutralizing response in SARS-CoV-2 naturally infected patients is important to estimate immune protection in this population and, possibly, to set shared guidelines.

Thanks to massive research efforts conducted since the beginning of the pandemics, multiple neutralizing antibodies have been characterized in terms of both affinity and epitope recognition. These studies revealed that distinct domains in the Spike protein are crucial for neutralization, namely, the RBD and the N-terminal domain (NTD) (9). Despite the proven role of RBD recognition in neutralization, recent work reported—though in a very limited number of patients—that the large majority of serum Spike IgG in the repertoire recognizes non-RBD epitopes (10). This observation suggests a broad response in terms of specificity in naturally infected individuals. Nevertheless, the actual breath of the IgG response in terms of epitope recognition across a cohort of naturally infected individuals is still unknown, and this aspect might be relevant to evaluate protection against different SARS-CoV-2 variants.

Here, we provide the characterization of SARS-CoV-2 antibody response in a cohort of 94 COVID-19 convalescent patients. To efficiently assess neutralization, we first developed and validated across a large number of samples a pseudotype-based neutralization assay. We evaluated the relationship between neutralization titers, disease severity, and recognition of Spike and RBD by serum IgG. Finally, we estimated the proportion of RBD-specific IgG across Spike-specific IgG. These results contribute to consolidate and expand our knowledge of humoral immunity in the context of COVID-19 natural infection.

## Results

### Cohort of COVID-19 Convalescent Patients

**Figure 1:**
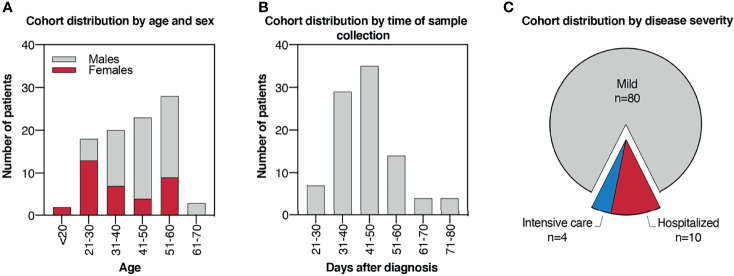
Cohort of COVID-19 convalescent patients. **(A)** Histogram plot indicating cohort distribution by age expressed in years and sex. **(B)** Histogram plot indicating cohort distribution by time of sample collection expressed as days after PCR**-**confirmed COVID-19 diagnosis. **(C)** Pie chart indicating cohort distribution by disease severity. Patients who did not require hospitalization were defined as “mild” cases, while hospitalized patients were defined as “severe” cases. Among the severe cases, patients who underwent intensive care were reported.

To evaluate the humoral response elicited by SARS-CoV-2 natural infection, we selected a cohort of recovered COVID-19 patients consisting of 94 individuals with PCR-confirmed SARS-CoV-2 infection. Infections occurred between February and April 2020, at the initial phase of the pandemic in Italy, so the population was presumably naïve for this virus prior to infection. The cohort includes 35 females and 59 males, with ages comprised between 19 and 64 years (**Figure 1A**). Serum was collected from patients in the cohort between 22 and 80 days after the diagnosis by molecular SARS-CoV-2 test, with most of the samples (83%) collected between 31 and 60 days after (**Figure 1B**). Among the 94 patients, 80 (85%) did not require hospitalization and were defined as mild cases, while 14 (15%) were hospitalized and were defined as severe cases. Among the severe cases, 4 patients were hospitalized in the intensive care unit, while the remaining 10 did not (**Figure 1C**).

### Development and Validation of a High-Throughput Neutralization Assay Based on rVSV

To characterize the humoral response elicited by SARS-CoV-2 natural infection, we sought to measure viral neutralization in serum samples from convalescent patients in our cohort. The classical neutralization assay carried out with live SARS-CoV-2 requires biosafety level 3 (BSL-3) facilities, which are typically less available than facilities with a lower biosafety profile. Moreover, the use of readouts that relies on non-automated procedures makes the assay laborious and time-consuming, limiting the number of samples that can be screened simultaneously. Work from different research groups carried out in the last months has proposed multiple pseudovirus-based assays as a proxy to evaluate SARS-CoV-2 neutralization (11–16). Nevertheless, not all platforms underwent a robust validation over a large number of samples.

**Figure 2:**
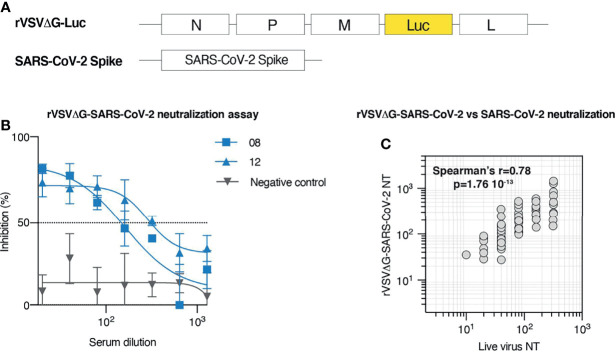
Development and validation of a high throughput neutralization assay based on rVSV. **(A)** Schematic representation of the elements employed to generate rVSVΔG-SARS-CoV-2 pseudotypes. rVSVΔG-Luc encodes for N, P, M, and L VSV proteins, but lacks G coding sequence, which has been replaced by the coding sequence of Luciferase. SARS-CoV-2 Spike construct encodes for SARS-CoV-2 glycoprotein. **(B)** rVSVΔG-SARS-CoV-2 neutralization assay displaying % of viral inhibition across 7 sample dilutions. Representative data (mean ± SD) of two samples from COVID-19 convalescent individuals (# 08; 12) and a prepandemic serum sample (negative control) are shown. **(C)** Correlation between serum neutralization titers obtained with rVSVΔG-SARS-CoV-2 pseudotypes and live SARS-CoV-2 across 60 randomly selected patients from our cohort.

To assess whether SARS-CoV-2 neutralization could be reliably measured in BSL-2 facilities and in a high throughput manner using a pseudovirus-based assay, we took advantage of a previously described replication-defective recombinant Vesicular Stomatitis Virus (rVSV) where the sequence encoding the viral glycoprotein G was replaced by the luciferase gene (rVSV**Δ**G-Luc) (**Figure 2A**). The presence of the Luc as a reporter allows to efficiently estimate viral infectivity based on a rapid luciferase assay (17). Moreover, Luc robust expression early upon infection allows to complete the assay in less than 1 day. To test this approach, we first generated rVSV**Δ**G-Luc pseudotyped with the SARS-CoV-2 Spike glycoprotein (rVSV**Δ**G-Luc-SARS-CoV-2) and then measured neutralization in 60 serum samples randomly selected from our patient cohort. Neutralization activity was expressed as neutralization titer, defined as the interpolated serum dilution producing a 50% reduction of virus infectivity. For each sample, residual infection was estimated across seven serum dilutions ranging from 1:20 to 1:1280, and the normalized % inhibition was calculated using as references prepandemic serum (0% inhibition) and signal obtained in uninfected cells (100% inhibition) (**Figure 2B**).

To validate this approach, we measured live virus neutralization on the same 60 samples initially tested for rVSV**Δ**G-Luc-SARS-CoV-2 neutralization. Analysis of the relationship between neutralization titers measured with live SARS-CoV-2 and with rVSV**Δ**G-Luc-SARS-CoV-2 indicates that the results obtained with these two approaches strongly correlate (Spearman’s r = 0.78; p < 0.0001; **Figure 2C**). This evidence supports the use of rVSV**Δ**G-Luc-SARS-CoV-2 neutralization assay to efficiently and safely measure neutralization titers in patients and extend previous validation of the rVSV-based platform, so far based on a more limited number of samples (12, 18).

### Neutralization Titer Broadly Differs Among COVID-19 Recovered Patients and Correlates With the Severity of Symptoms

**Figure 3:**
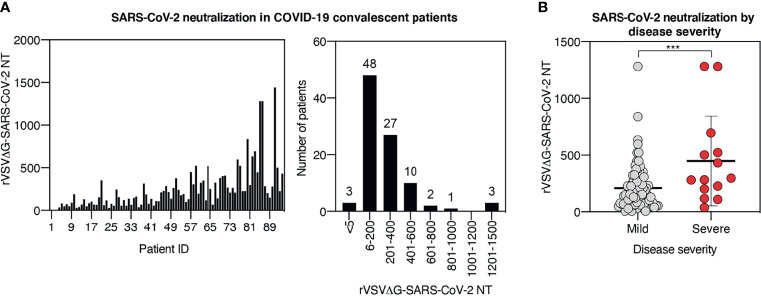
Neutralization titer broadly differs among COVID-19 recovered patients and correlates with the severity of symptoms. **(A)** rVSVΔG-SARS-CoV-2 neutralization titers in serum samples from the cohort. Results are displayed by patient ID (left panel) and in histogram plot indicating cohort distribution by neutralization titers (right panel). **(B)** Plot displaying rVSVΔG-SARS-CoV-2 neutralization titers in patients stratified by disease severity. Each symbol represents one patient. For each group, the mean ± SD is indicated; *** indicates p < 0.001.

Based on the strong correlation between results obtained with live virus neutralization assay and rVSV**Δ**G-Luc-SARS-CoV-2 neutralization assay, we performed the pseudotype-based neutralization assay in all samples from our cohort. Serum samples displayed a broad range of neutralization titers ranging from <5 to 1,442, with the vast majority of samples (n = 85, 90%) showing a neutralization titer comprised between 6 and 600. Only 6% of the samples displayed a very high neutralization titer (≥601), while in 3% of the cases, neutralization was absent (titer < 5) (**Figure 3A**).

The humoral response can be influenced by a plethora of factors, including antigen load, antigen persistence, innate immune activation, and genetic background, just to mention a few. Although the influence of each of these factors has not been fully addressed in human COVID-19 humoral response, accumulating observations suggest that humoral response differs in patients with distinct clinical disease progression (19–23). To assess the relationship between disease severity and neutralization titer in our cohort, we stratified patients based on disease severity. Analysis of neutralization activity in severe versus mild cases showed significantly higher neutralization titers in severe cases than in mild cases (mean neutralization of 447 and 208, respectively, p < 0.001) (**Figure 3B**), indicating that the majority of hospitalized patients mounted a more effective neutralizing response compared with the less severe cases.

Collectively, these data confirm that the vast majority of COVID-19 recovered patients developed neutralizing antibodies against SARS-CoV-2 and show that the potency of the neutralizing response varies broadly across patients. Moreover, our results validate the observations that patients experiencing a more severe disease are more likely to develop a highly neutralizing humoral response.

### Convalescent Patient Serum Contains Variable Titers of Anti-Spike and Anti-Receptor-Binding Domain IgG Antibodies That Correlate With Neutralization Potency

**Figure 4:**
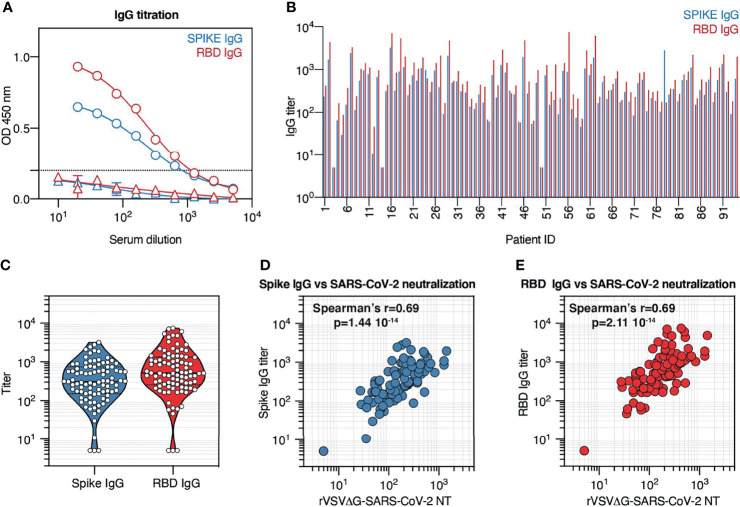
Convalescent patient serum contains variable titers of anti-Spike and anti-receptor-binding domain (RBD) IgG antibodies that correlate with neutralization potency. **(A)** Spike and RBD IgG titration by ELISA across 10 serum sample dilutions. Representative data (mean ± SD) of a COVID-19 convalescent individual (circles) and a prepandemic serum sample (triangles) are shown. **(B)** Spike and RBD IgG titers in serum samples from the cohort. Results are displayed by patient ID. **(C)** Violin plot indicating Spike and RBD IgG titer distribution across the cohort. Each symbol represents one patient. **(D)** Correlation between serum neutralization titers obtained with rVSVΔG-SARS-CoV-2 pseudotypes and Spike IgG titer across all patients from our cohort. **(E)** Correlation between serum neutralization titers obtained with rVSVΔG-SARS-CoV-2 pseudotypes and RBD IgG titer across all patients from our cohort.

Neutralization potency varied substantially across recovered COVID-19 patients. Differences in neutralization potency depend on multiple factors, namely, the concentration, specificity, and affinity of antibodies elicited by infection. To assess the specificity and the relative abundance of SARS-CoV-2 antibodies, and their correlation with neutralization potency, serum samples from COVID-19 convalescent patients were tested by ELISA against full Spike protein consisting of both S1 and S2 domains, and against Spike RBD (**Figure 4A**). Since serum samples were collected at least 3 weeks after COVID-19 diagnosis, we focused our attention on IgG rather than IgM, since IgM is expected to decline more rapidly (24, 25). The results showed variable levels of anti-Spike and anti-RBD IgG in convalescent individuals (**Figure 4B**). In three samples, the antibody level, against both Spike and RBD, was below the cutoff value and was assigned a titer of 5. To assess if these patients did not develop Spike or RBD antibodies at all, or if other isotypes different from IgG were present, we tested the corresponding samples for the presence of Spike and RBD IgM and IgA antibodies. All three patients showed low IgM titer (**Figure S1A**), while only one had detectable IgA (**Figure S1B**), indicating that the patients did experience SARS-CoV-2 infection but failed to mount an effective IgG response. The rest of the samples showed an ELISA IgG titer comprised between 10 and 3,208 for Spike and 10 and 7436 for RBD, with median titers in the assessed population of 313 and 515, respectively (**Figure 4C**).

To determine the relationship between antibody recognition of Spike protein, and in particular of the RBD, and neutralization potency in the serum of recovered COVID-19 patients, we compared Spike and RBD IgG titers with neutralization titers across our cohort (**Figures 4D, E**). Collectively, we observed that both Spike and RBD IgG titers positively correlate with neutralization titers (Spearman’s r = 0.69; p < 0.001), supporting previous observations that neutralization activity relies on Spike and RBD recognition and that Spike and RBD IgG titers can be employed to predict neutralization potency (23).

### Spike IgG Antibodies Generated After Natural COVID-19 Infection Are Not Polarized Toward the Receptor-Binding Domain Region

**Figure 5:**
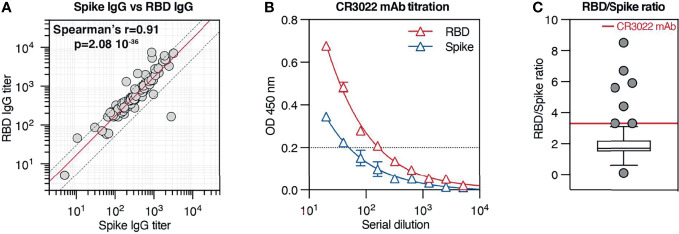
Spike IgG antibodies generated after natural COVID-19 infection are not polarized toward the receptor-binding domain (RBD) region. **(A)** Correlation between Spike IgG titer and RBD IgG titers across all patients from our cohort. The red solid line indicates the mean RBD/Spike titer ratio (1.7), while the two dotted lines indicate the SD ( ± 1.2). **(B)** Spike and RBD IgG titration (mean ± SD) by ELISA across 9 serial dilutions of the anti-RBD monoclonal Ab CR3022. **(C)** Ratio between RBD and Spike IgG titers across all patients in the cohort. Results are in box-and-whiskers plot (Tukey) indicating cohort distribution (right panel, only individual data outside from the whiskers are displayed with a symbol). The red line indicates the RBD/Spike titer ratio relative to CR3022 mAb (3.3).

It has been established that RBD is a crucial target of neutralizing Abs. Nevertheless, it is not clear whether, in the context of natural COVID-19 infection, RBD recognition is similarly represented among anti-Spike IgG responses across different patients. To investigate this aspect, we compared Spike IgG titers and RBD IgG titers from serum samples from our cohort and observed that Spike and RBD IgG titers strongly correlate with each other (Spearman’s r = 0.91; p < 0.001) and exhibit a mean RBD/Spike titer ratio of 1.7 (SD ±1.2) (**Figure 5A**). Of all patients in our cohort, only 8 (8.5%) displayed an RBD/Spike titer ratio significantly different from the mean. This result indicates that in the majority of the samples, RBD and Spike responses develop in similar relative proportions.

Next, we wondered if antibodies recognizing Spike protein are preferentially targeting the RBD or if other domains dominate the response. Interestingly, recent work from Voss and colleagues (10) based on the proteomic analysis of the IgG repertoire from four convalescent COVID-19 patients indicates that the Spike IgG response is directed predominantly (>80%) against epitopes outside the RBD. To estimate the relative contribution of RBD recognition among IgG specific for Spike, we took as a reference the purified monoclonal antibody CR3022, which binds potently to SARS-CoV-2 RBD (26). We titrated CR3022 against both RBD and Spike in identical test conditions used for serum samples and observed on average a 3.3 times higher titer on RBD-coated plates than on Spike-coated plates (**Figure 5B**), consistently with the different molar concentration of ligands used for coating. We reasoned that, in our experimental conditions, an RBD/Spike titer ratio of 3.3 would be measured when all Spike IgG in the sample are targeting RBD, while lower ratios would indicate a less prevalent RBD recognition. When comparing CR3022 mAb RBD/Spike titer ratio with the ratios displayed by patients in our cohort, we observed that the large majority of the patients displayed a ratio lower than 3.3, with actually 77% of the patients displaying a ratio comprised between 1 and 2.5 (**Figure 5C**). Only a few tested individuals presented a ratio similar to the one exhibited by CR3022 mAb (3.1, 3.3, and 3.3) or higher (4.4, 6.7, 5.6, 5.9, and 8.5), carrying presumably antibodies directed predominantly against RBD and/or antibodies that recognize poorly the RBD motif in the context of the Spike conformation used for the assay. Interestingly, only one individual showed a much higher Spike titer than RBD titer (2782 Spike/166 RBD, ratio 0.1), suggesting that the immunological response was focused against regions located on the Spike protein different than RBD. The fact that the majority of antibodies contained in this sample were directed against regions outside of the RBD can explain why the serum does not have very high neutralizing activity (live virus neutralization titer 80). Despite the fact that this assay does not have the ability to estimate the specificity of individual antibodies and their relative prevalence in the serum, the modest variation in RBD/Spike titer ratio across our cohort suggests that overall RBD recognition is present in a comparable manner in the majority of COVID-19 patients. Finally, the observation that most patient samples display an RBD/Spike titer ratio lower than CR3022 mAb is consistent with a response not polarized toward the RBD, suggesting that other epitopes are targeted in the context of naturally occurring COVID-19 infection.

## Discussion

This work was conducted on a cohort of patients comprising 94 COVID-19 convalescent individuals who experienced infection between February and April 2020, during the first peak of COVID-19 incidence in Padua region, Italy. In all cases, SARS-CoV-2 infection was confirmed by detection of viral genetic material, and serum samples were collected 22 to 80 days after positive molecular testing. Based on the timing of SARS-CoV-2 infection, and the extremely low incidence in the Italian population of SARS-CoV-2 phylogenetically related viruses, as SARS-CoV and Middle East respiratory syndrome coronavirus (MERS-CoV), the population was likely naïve for SARS-CoV-2 itself and for viruses that might induce a SARS-CoV-2 cross-reactive response. Together, these characteristics allowed for the analysis of the humoral response elicited by naturally occurring primary SARS-CoV-2 infection, in a population that did not experience yet antigenic overlapping immune responses, for instance, vaccination.

To rapidly and safely measure SARS-CoV-2 neutralization, we established and validated across a large number of samples an rVSV**Δ**G-Luc-based pseudotype assay. This assay can be conducted in a BSL-2 facility, and thanks to the fast expression of Luciferase, results can be obtained in just 1 day. Importantly, the high correlation between rVSV**Δ**G-Luc-SARS-CoV-2 and live virus neutralization titers supports the use of the rVSV platform for SARS-CoV-2 neutralization and entry studies.

The analysis of serum neutralization potency in our cohort indicated large variations across SARS-CoV-2 convalescent individuals, consistently with other reports (27). Serum collection in our cohort occurred at different times after molecularly confirmed COVID-19 diagnosis. Nevertheless, due to the reported longevity of the IgG response in COVID-19 recovered patients (5), the observed variations can be only minimally imputed to differences in sample collection times. Rather, our analysis shows how neutralization potency is significantly associated with disease severity, with hospitalized patients exhibiting higher neutralization titers compared with patients with milder symptoms. This association has been independently reported by other groups (27–30) and can be explained by the possibility that uncontrolled viral spread leads to increased pathology, exacerbated inflammation, and also increased viral antigen load, which will favor humoral response. Interestingly, it has been reported that in hospitalized patients, the development of RBD IgG was associated with improved patient survival, supporting a beneficial role of humoral response in the clearance of infection (31).

Based on the modeled relationship between neutralization titers and protection in SARS-CoV-2 infection (32) and on the observed variability in neutralization titers in our cohort, it is expected that different SARS-CoV-2 recovered patients exhibit different susceptibility to secondary infection, and in particular that patients who were infected by SARS-CoV-2 but experienced mild disease might be more vulnerable to reinfection than patients with more severe disease. These differences in expected protection fully support current public health guidelines encouraging vaccination of recovered COVID-19 individuals to achieve robust neutralization and protection.

The measurement of Spike and RBD IgG titers by ELISA in serum samples revealed broadly variable levels across patients from our cohort, which nevertheless correlated significantly with neutralization potency. This observation provides additional evidence that recognition of Spike, and in particular of RBD, is crucial to achieve neutralization and supports the quantification of RBD IgG for the prediction of neutralization potency in COVID-19 recovered and vaccinated individuals.

Finally, we investigated if in our cohort the anti-Spike IgG response was polarized toward the RBD, or if other specificities were present. To estimate the proportion in each patient of anti-RBD IgG across total anti-Spike IgG, we calculated the ratio between RBD and Spike IgG titers and compared it to the ratio obtained by titrating the CR3022 monoclonal Ab, an antibody recognizing the RBD region on SARS-CoV-2 Spike. Interestingly, while Spike and RBD IgG titers were highly variable across the studied population, their relative proportion was consistent in the majority of the patients, implying that RBD recognition is a conserved feature of the humoral response against SARS-CoV-2. We also observed that the ratio between RBD and Spike IgG titers in the vast majority of the patients was lower (≤50% in 65% of the patients) than the ratio measured for CR3022 mAb, suggesting that RBD-targeting antibodies represent only a modest proportion of Spike-specific IgG. These data support evidence recently reported from other groups using different approaches. In-depth proteomic analysis of Spike IgG lineage in four donors revealed that the majority of IgG in the analyzed samples target epitopes outside the RBD (10); moreover, removal of RBD IgG from polyclonal serum only modestly affects Spike recognition (33). Taken together, these observations indicate that the humoral response elicited by natural COVID-19 infection is not polarized toward a single Spike domain but rather directed to different epitopes, with possible beneficial effects toward protection against distinct SARS-CoV-2 variants. Additional characterization of antibodies elicited by SARS-CoV-2 infection or vaccination in terms of epitopes recognized and neutralization ability will be relevant to predict protection against arising novel SARS-CoV-2 variants.

## Methods

### Patient Samples

Serum samples used in the study were obtained on average 6 weeks (SD 2 weeks, range 22–80 days) after PCR test confirming SARS-CoV-2 infection. Specimens were heat-inactivated for 30 min at 56°C and stored at −20°C. Prepandemic serum samples collected in 2017 were used as a negative control.

### Plasmids

Expression vectors containing the coding sequences of the SARS-CoV-2-stabilized, soluble Spike ectodomain (pCAGGS-Spike) and of the RBD (pCAGGS-RBD) were kindly provided by Dr. Florian Krammer (Icahn School of Medicine at Mount Sinai) and were described previously (34). The vector encoding the full-length SARS-CoV-2 Spike employed for pseudotype generation was produced under HHSN272201400008C and obtained through BEI Resources, NIAID, NIH (BEI Resources, Manassas, VA, USA; Cat. # NR-52310).

### Cells

FreeStyle™ 293-F Cells (Gibco, Grand Island, NY, USA; Cat. # R79007) were cultured in FreeStyle™ 293 Expression Medium (Gibco, Cat. # 12338018) and maintained at 37°C, 8% CO_2_, 80% humidity, on a shaker platform rotating at 130 rpm.

Human embryonic kidney 293 cells containing the SV40 T-antigen (HEK293T) and African green monkey kidney cells (VERO) were cultured in Dulbecco’s Modified Eagle’s Medium High Glucose (DMEM; EuroClone, Pero, Italy; Cat. # ECB7501L × 10) supplemented with 10% heat-inactivated fetal bovine serum (FBS; Gibco, Cat. # 10270), 1% Penicillin/Streptomycin (P/S; EuroClone, Cat. # ECB3001D), and 1% GlutaMAX (Gibco, Cat. # 35050-038) and maintained at 37°C, 5% CO_2_, 80% humidity.

### SARS-CoV-2 Neutralization Assay

Twofold dilutions of serum samples were made starting at a 1:10 dilution, distributed to 96 well plates, mixed 1:1 with a SARS-CoV-2 virus solution containing one hundred 50% tissue culture infectious dose (TCID50), and incubated for 1 h at 37 °C, in a 5% CO_2_-humidified atmosphere. After incubation, VERO cell suspension, previously detached in DMEM 6% FBS, was added to each well and further incubated at 37 °C. At 72 h of incubation, the microplates were treated with 5% formaldehyde 40% Gram’s crystal violet, incubated for 30 min, washed, and allowed to dry; and the absorbance was read at 595 nm. The highest serum dilution showing an optical density (OD) value equal to or greater than 90% of the control serum was considered as the neutralization titer.

### rVSVΔG-Luc-SARS-CoV-2 Pseudotype Production

The rVSV in which the glycoprotein (G) gene has been deleted (VSVΔG) and replaced with firefly luciferase (Luc) has been originally described by Whitt (17). To generate rVSVΔG-SARS-CoV-2, HEK 293T cells were transfected with 12 µg of pCAGGS vector encoding the full-length SARS-CoV-2 Spike using calcium phosphate transfection method. At 36 h post transfection, cells were infected with rVSVΔG-Luc-VSVG as described (17). Twenty-four hours post infection, the supernatant was collected, centrifuged, aliquoted, and frozen at −80°C. The titer of generated rVSV**Δ**G-Luc-SARS-CoV-2 pseudotype stock was determined by preparing twofold dilution in complete medium in duplicate and plating onto VERO cells pre-seeded the day before on a 96-well plate at the concentration of 0.2 × 10^5^ per well. Twenty-four hours later, the ONE-Glo Luciferase Assay System substrate/lysis solution was added (Promega, Madison, WI, USA; Cat. # E6120), and luminescence was measured using PerkinElmer (Waltham, MA, USA) plate reader (VICTOR™ X4).

### rVSVΔG-SARS-CoV-2 Pseudotype Neutralization Assay

Twenty-four hours before sample preparation, VERO cells were plated into 96-well culture plates (0.2 × 10^5^/well). The following day, twofold serial dilutions of serum samples in duplicates (60 µl/well) were prepared in DMEM high-glucose 5% FBS complete medium and mixed with an equal volume of medium containing rVSVΔG-SARS-CoV-2 pseudotype at the concentration of 4 × 10^6^ RLU/ml (0.2 × 10^6^ RLU/50 µl). Dilution plates were then incubated for 1 h at 37°C. The serum–pseudotype mixture measuring 100 µl from each well was transferred to the corresponding well of cell culture plate containing pre-seeded VERO and incubated 24 h at 37°C, 5% CO_2_, 80% humidity.

After incubation, 100 µl of the ONE-Glo Luciferase Assay System substrate/lysis solution was added into each well, incubated for 2 min at room temperature (RT) to allow complete cell lysis, and transferred to corresponding well onto a white, 96-well plate for luminescence readout. Luminescence was measured using a PerkinElmer plate reader (VICTOR™ X4). Percent neutralization was normalized considering uninfected cells as 100% neutralization and cells infected but not treated with serum as 0% neutralization. IC_50_ titers were determined using a non-linear, sigmoidal, 4PL function in Prism v8 (GraphPad, La Jolla, CA, USA).

### SARS-CoV-2 Spike and Receptor-Binding Domain Expression and Purification

Expression and purification of His tagged SARS-CoV-2 Spike and RBD were carried out according to protein expression and purification procedure described in (35) with some modifications and is described in detail below.

Proteins were expressed in FreeStyle™ 293-F cells. The day before, transfection cells were passed to fresh FreeStyle™ 293 expression medium at a concentration of 0.6 × 10^6^ cell/ml in 250-ml final volume into 1-L baffled culture flask. On the transfection day, 250 µg of plasmid DNA encoding Spike or RBD was diluted in 5 ml of OptiMEM (Thermo Fisher, Waltham, MA, USA; Cat. # 31985070), while in a separate vial, 0.5 ml of 1 mg/ml stock solution polyethylenimine (PEI; PolySciences, Warrington, PA, USA; Cat. # 23966-1) was diluted in 4.5 ml of OptiMEM. Solutions were incubated for 15 min, RT, and then PEI solution was added to the DNA solution, mixed gently, and incubated for 15 min, RT. The DNA : PEI solution was added to the cell culture in a dropwise manner. Transfected cells were incubated for 4 days on an orbital shaker platform rotating at 90 rpm at 37°C, 8% CO_2_, 80% humidity.

Culture supernatants were collected by centrifugation at 4,000 ×*g*, 20 min, 4°C, filtered using a 0.2-µm filter, and buffered by adding 1/10 vol. of 10× phosphate-buffered saline (PBS), pH 7.4. Ni Sepharose^®^ excel affinity media (GE Healthcare, Chicago, IL, USA; Cat. # 17-3712-01) measuring 5 ml was washed 3 times with PBS pH 7.4 and added to each culture supernatant. Solutions were incubated in RT for 2 h on an orbital shaker. Culture supernatant with resin was loaded on columns, and the resin was washed with wash buffer (50 mM of NaH_2_PO_4_, 300 mM of NaCl, and 20 mM of imidazole, pH 7.4). Recombinant proteins were eluted with elution buffer (50 mM of NaH_2_PO_4_, 300 mM of NaCl, and 235 mM of imidazole, pH 7.4); then eluates were repeatedly diluted in PBS and concentrated using Amicon Ultra Centrifugal Filter Units (Millipore, Billerica, MA, USA; Cat. # UFC9010) to remove imidazole. Protein purity and concentration were estimated using 280-nm absorbance and sodium dodecyl sulfate–polyacrylamide gel electrophoresis (SDS-PAGE) with bovine serum albumin (BSA) standards. Purified antigens were aliquoted and stored at −20°C.

### SARS-CoV-2 Spike and Receptor-Binding Domain ELISA

ELISAs performed in this study were adapted from previously established protocols (34, 36). High protein binding, half area, 96 well microplates (Corning^®^, New York, NY, USA; Cat. # 3690) were coated O/N at 4°C with 30 µl of 2 µg/ml of RBD or Spike protein. Plates were washed with PBS 0.1% Tween 20 (PBS-T) and blocked with 120 μl of PBS-T + 3% non-fat dry milk (NFDM) for 2 h at RT. Samples in duplicates were serially diluted in PBS-T + 1% NFDM. Plates were incubated with 60 µl/well of serum dilutions for 2 h at RT, washed with PBS-T, and incubated 1 h, RT, with 60 µl/well of horseradish peroxidase (HRP)-conjugated goat anti-human IgG secondary antibody (Bethyl Laboratories, Montgomery, TX, USA; Cat. # A80-119P) at 1:75,000 dilution in PBS-T + 1% NFDM. Plates were washed, and 60 µl/well of SIGMAFAST™ OPD peroxidase substrate solution (SIGMA, St. Louis, MO, USA; Cat. # P9187-50SET) was added. After exactly 10 min, the reaction was stopped by the addition of 30 µl/well of 3N of HCl, and absorbance was measured at 490 nm using a PerkinElmer reader (VICTOR™ X4). Background OD (defined as the OD measured in wells not incubated with any serum) was subtracted from the OD measured in sample wells. Based on the absorbance exhibited by pre-pandemic serum samples, an OD 0.2 was determined as a cutoff value. For each serum sample, the titer (defined as the dilution where the sample shows an OD = cutoff value) was determined using a non-linear, asymmetric fitting (Prism v8, GraphPad) of the measured and background-corrected OD reported across 10 serial dilutions.

### Statistical Analysis

The statistical significance of the difference in mean neutralization titer of mild vs. severe patients was assessed by an unpaired t-test. Correlation analysis was performed by calculating Spearman’s r. All statistical analyses were carried out using GraphPad Prism software.

## Data Availability Statement

The raw data supporting the conclusions of this article will be made available by the authors, without undue reservation.

## Ethics Statement

The studies involving human participants were reviewed and approved by the Ethical Committee for Clinical Research of the province of Padua. The patients/participants provided their written informed consent to participate in this study.

## Author Contributions

Conceived and designed the study: AM, GS, AR, and GP. Collected the clinical samples for the study: MR. Performed the experiments: AM and MP. Analyzed the data: AM and GP. Contributed key material and support for pseudotype production: CS. Wrote the paper: AM and GP. All authors contributed to the article and approved the submitted version.

## Funding

This work was supported by CARIPARO Foundation COVID-19 research grant (AR and GP).

## Conflict of Interest

The authors declare that the research was conducted in the absence of any commercial or financial relationships that could be construed as a potential conflict of interest.

## Publisher’s Note

All claims expressed in this article are solely those of the authors and do not necessarily represent those of their affiliated organizations, or those of the publisher, the editors and the reviewers. Any product that may be evaluated in this article, or claim that may be made by its manufacturer, is not guaranteed or endorsed by the publisher.

## Supplementary Material

Supplementary Figure 1 IgM and IgA titers in IgG negative COVID-19 patients from the cohort. **(A)** Spike and RBD IgM titers in patients #03, 14 and 50 who showed negative Spike and RBD IgG titers. **(B)** RBD IgA titers in patients #03, 14 and 50.
