# Supplementary figures and images for: Neutralizing Antibody Responses to SARS-CoV-2 in Recovered COVID-19 Patients Are Variable and Correlate With Disease Severity and Receptor-Binding Domain Recognition
Source: Front Immunol. 2022 Jan 31;13:830710. doi: 10.3389/fimmu.2022.830710 (PMC8841804; doi:10.3389/fimmu.2022.830710)

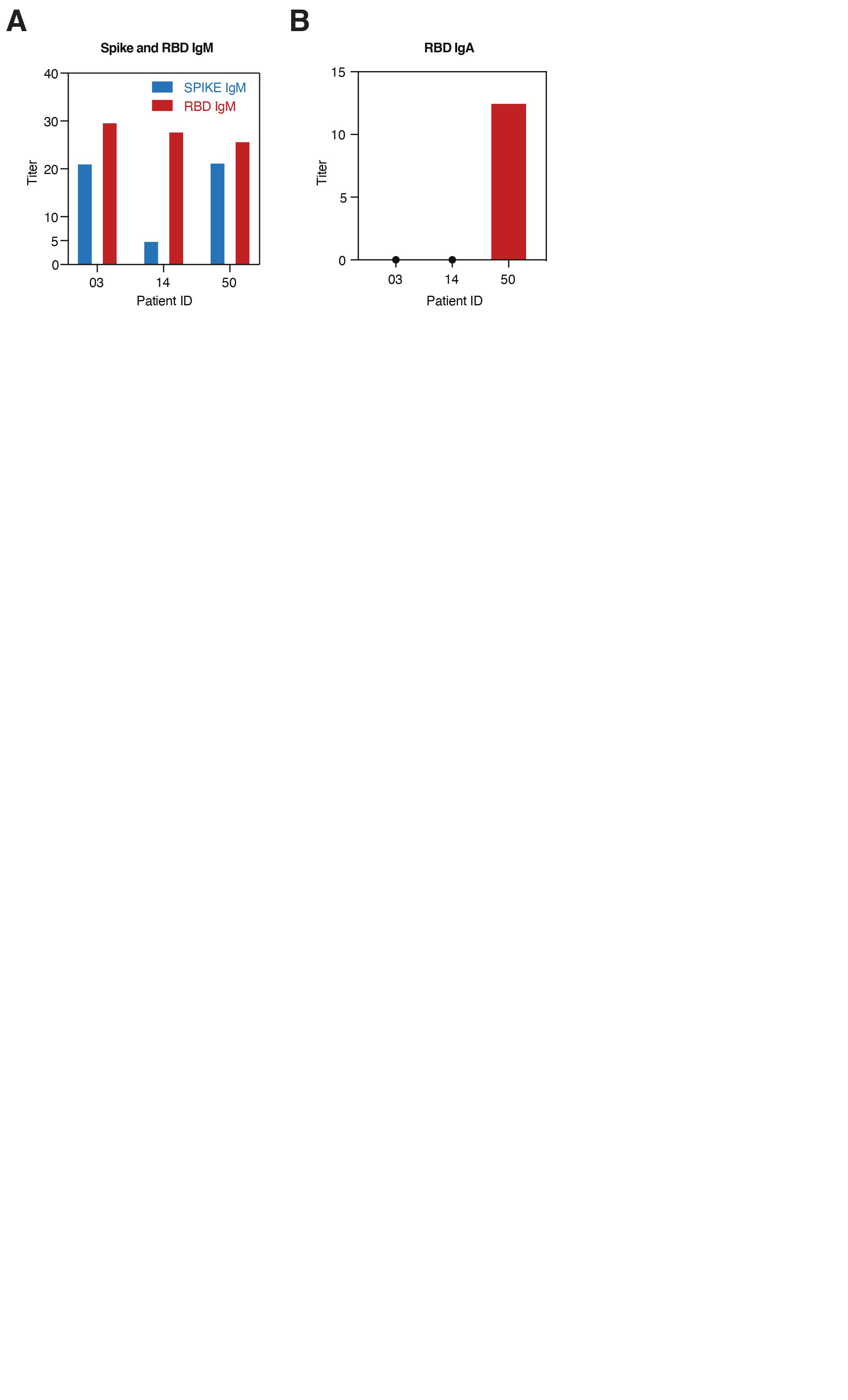

Supplement: Supplementary Figure 1 — IgM and IgA titers in IgG negative COVID-19 patients from the cohort. (A) Spike and RBD IgM titers in patients #03, 14 and 50 who showed negative Spike and RBD IgG titers. (B) RBD IgA titers in patients #03, 14 and 50. [file Image_1.jpeg]
